# Noncoding RNAs in cancer immunity: functions, regulatory mechanisms, and clinical application

**DOI:** 10.1186/s12943-020-01154-0

**Published:** 2020-03-02

**Authors:** Le Zhang, Xiaonan Xu, Xiulan Su

**Affiliations:** 1 Clinical Medical Research Center of the Affiliated Hospital, Inner Mongolia Medical University, 1 Tong Dao Street, Huimin District, Hohhot, 010050 Inner Mongolia China; 2 Department of Molecular Oncology, H. Lee Moffitt Cancer Center and Research Institute, Tampa, Florida, FL 33612-9497 USA

## Abstract

It is well acknowledged that immune system is deeply involved in cancer initiation and progression, and can exert both pro-tumorigenic and anti-tumorigenic effects, depending on specific microenvironment. With the better understanding of cancer-associated immune cells, especially T cells, immunotherapy was developed and applied in multiple cancers and exhibits remarkable efficacy. However, currently only a subset of patients have responses to immunotherapy, suggesting that a boarder view of cancer immunity is required. Non-coding RNAs (ncRNAs), mainly including microRNAs (miRNAs) and long noncoding RNAs (lncRNAs), are identified as critical regulators in both cancer cells and immune cells, thus show great potential to serve as new therapeutic targets to improve the response of immunotherapy. In this review, we summarize the functions and regulatory mechanisms of ncRNAs in cancer immunity, and highlight the potential of ncRNAs as novel targets for immunotherapy.

## Introduction

Immune system attacks organisms and substances that invade body systems and cause disease. The two main classes of the immune system are the innate immune system and the adaptive immune system [1]. The innate immune system is the first line of defense against foreign pathogens, and the adaptive immune system provides highly specialized processes eliminating pathogens [2, 3]. Importantly, in addition to defending exogenous pathogens, immune system can also eliminate cancer cells. Therefore, in the past few decades, researchers and physicians dedicated to efficiently activate immune system for better fighting cancer, and such treatment is called “Immunotherapy”. Indeed, Immunotherapy exhibits remarkable and durable efficacy and has been established as the fourth treatment pillar of cancer therapy [4]. However, only a minority of patients have incredible responses to immunotherapy. Therefore, an improved understanding of the immune system and how it interacts with cancer cells will be helpful to develop effective therapeutic strategies for cancer immunotherapy that are on the horizon hold extraordinary promise for the future. Increasing evidence has identified that ncRNAs are active participants in multiple stages of tumor immunity [5–8]. ncRNAs, including miRNAs, lncRNAs, and circular RNAs (circRNAs), regulate diverse cellular processes in development and diseases through a variety of gene-regulation mechanisms [9]. A better understanding of ncRNA-mediated regulation of cancer immunity will provide novel targets for the development of new therapeutic strategies. Therefore, additional studies are needed to further uncover the roles of ncRNAs in cancer immunity and provide new insights into the diagnosis and immunotherapeutic treatment of cancer. In this review, we summarize and discuss the latest studies on the functions and regulatory mechanisms of ncRNAs in cancer immunity, highlight and clarify potential roles of ncRNAs as targets for immunotherapies, and share and provide future perspectives for the clinical application of ncRNA-based therapies.

## Development of cancer immunology

Cancer immunology was discovered by William Coley who speculated that a strep infection had reversed cancer growth in 1891 [10]. Over time, many studies have confirmed that homograft rejection could reject transplanted tumors [11–13]. Based on this viewpoint, in 1970 Macfarlane Burnet developed and systematically elucidated the first important theory of oncoimmunology, called immune surveillance [14]. In 1970s, A number of following studies discovered natural killer (NK) cells and dendritic cells (DCs), which was a major advance in our understanding of the immune responses [15–17]. In 2002, Robert Schreiber and Lloyd Old refined the cancer immunosurveillance hypothesis to describe cancer immunoediting which incorporated three different potential outcomes: elimination, equilibrium, and escape and continuously refined this hypothesis from 2002 to 2014 [18–21].

Notably, Daniel Chen and Ira Mellman proposed the cancer-immunity cycle in 2013.They systematically elucidated the biological process of the anticancer immune response. The cancer-immunity cycle is a series of stepwise events that can be divided into seven major steps: (1) release of cancer cell antigens; (2) cancer antigen presentation; (3) priming and activation of effector T cell responses; (4) trafficking of T cells to tumors; (5) infiltration of T cells into tumors; (6) recognition of cancer cells by T cells; and (7) killing of cancer cells. Importantly, overcoming the negative feedback mechanisms of the cancer-immunity cycle might be the best approach for clinical development of onco-immunology-based strategies [22].

## Functions of ncRNAs in immunity

ncRNAs are a class of RNA molecules that do not code for proteins. Importantly, the majority of the genome is composed of ncRNAs, which account for 98% [23]. They can be mainly classified into miRNAs, lncRNAs, and circRNAs [24]. miRNAs are 20–25 nucleotides in length and mediate posttranscriptional silencing of specific target genes [23]. They have been the most studied ncRNAs since they were first described in *Caenorhabditis elegans* in 1993 [25]. Another type of ncRNAs is lncRNAs, which were once considered “garbage sequences” that accumulated during the evolutionary process and were not worth much attention. However, increasing evidence has shown that the roles of lncRNAs in physiological functions in gene regulation are complicated and significant [26]. circRNAs, a novel type of ncRNA, have been found to be involved in many physiological processes and diseases, including different cancers and autoimmune diseases [27–29]. Here, we will focus on the well-established role of miRNAs and on the emerging roles of lncRNAs and circRNAs, which all contribute to modulation of tumorigenesis and antitumor immunity.

### Roles of ncRNAs in innate immunity

**Fig. 1:**
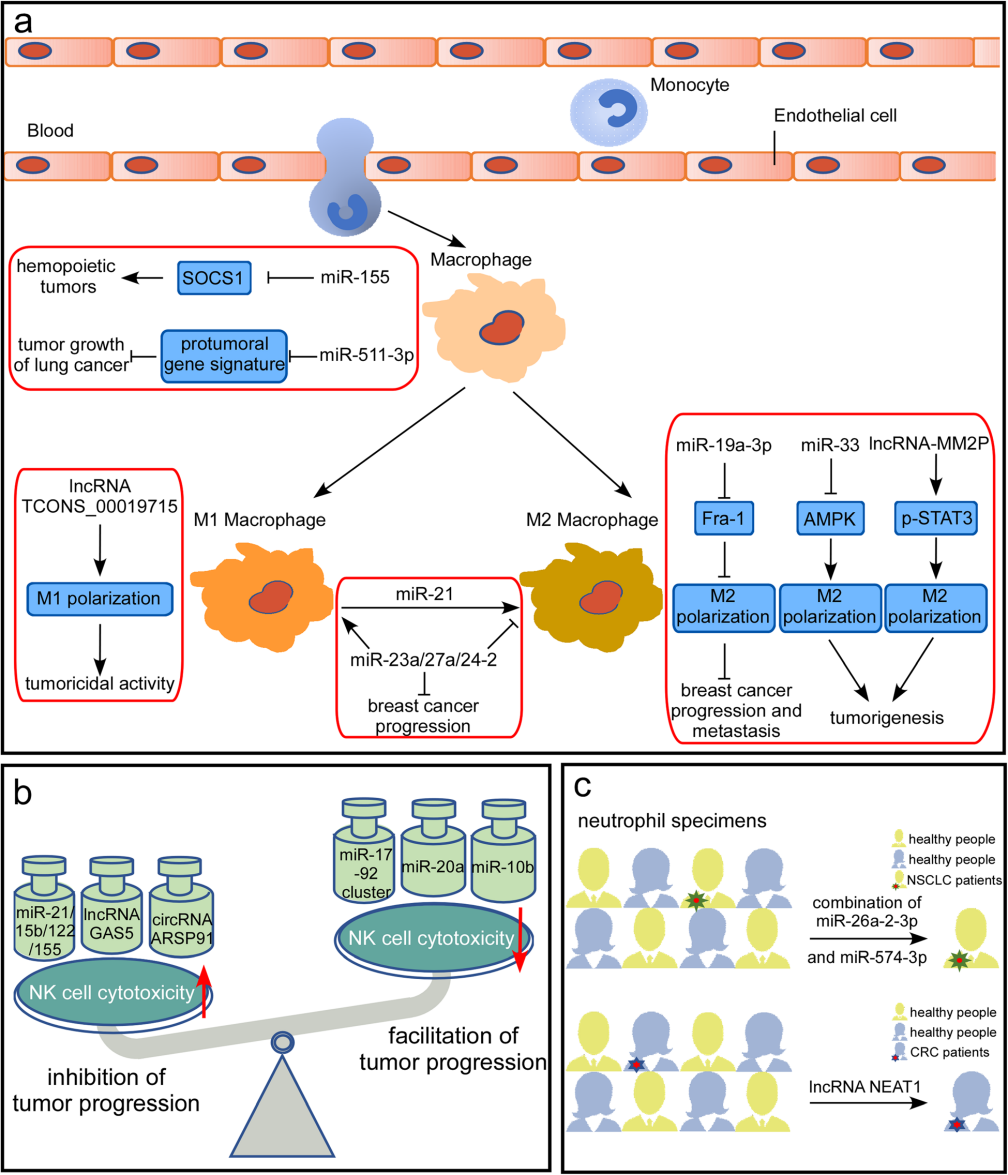
ncRNAs involved in innate immune regulation in cancer. **a** Studies have identified and highlighted the significance of ncRNAs in macrophage development and function. A visible representation of the ncRNAs involved in macrophages is provided. **b** Increasing evidence indicates that ncRNAs have important roles in NK cell biology in the contexts of development, inflammation, and tumor surveillance. The most relevant ncRNAs are included. **c** Examples of ncRNAs that differentiate neutrophils of cancer patients from those of cancer-free controls are shown, which indicates that ncRNAs modulate neutrophil functions and cancer pathogenesis

The innate immune system is the first line of defense against foreign pathogens, which vary extensively in their molecular composition, structure, and life cycle. Innate immunity consists of physical barriers, effector cells, pattern recognition mechanisms and humoral mechanisms [30, 31]. Among these components, effector cells, including macrophages, natural killer cells and neutrophils, play important roles in the innate immune response [32]. ncRNAs are critical in regulating these effector cells (Fig. 1).

#### Macrophages

Macrophages are produced through the differentiation of monocytes that have migrated from the bloodstream into any tissue in the body. They are responsible for eliminating harmful materials such as foreign substances, cellular debris and cancer cells. Studies have identified and highlighted the significance of ncRNAs in macrophage development and function (Fig. 1a). Wang et al. reported that miR-155 targeted SOCS1 in macrophages, which promoted type I IFN signaling in antiviral innate immunity and might contribute to the pathogenesis of many human hemopoietic tumors with overexpressed miR-155 [33]. miR-511-3p, an intronic miRNA, was described to downregulate the protumoral gene signature of MRC1+ TAMs and inhibit tumor growth [34]. This study revealed a role for miR-511-3p as a gatekeeper of the protumorigenic activity of Tumor-associated macrophages (TAMs). Considering that macrophage polarization is an important component of many disease states including cancer [35, 36], many studies have been performed to identify the role of ncRNAs in macrophage polarization. M1 macrophage polarization generally promotes Th1-type inflammatory responses and strong microbicidal and tumoricidal activities [35], while M2 macrophage polarization is essential for their function in immunological tolerance, which may promote tumorigenesis [37]. By utilizing lncRNA microarray, Huang et al. found that 9343 lncRNAs were deregulated in proinflammatory macrophages and that 4592 lncRNAs were deregulated in anti-inflammatory macrophages. Among these deregulated lncRNAs, lncRNA TCONS_00019715 might play a critical role in promoting macrophage polarization to the M1 phenotype, which enhances tumoricidal activities [35]. However, ncRNAs, such as miRNA-19a-3p, miR-33 and lncRNA-MM2P, regulate tumorigenesis by affecting M2 macrophage polarization. miRNA-19a-3p inhibited breast cancer progression and metastasis by inducing macrophage polarization through downregulating expression of the proto-oncogene Fra-1 [38]. By targeting the energy sensor AMPK, miR-33 mediates M2 polarization [39]. Based on lncRNA microarray assays, Cao et al. identified lncRNA-MM2P and revealed its role in macrophage-promoted tumorigenesis [37]. Knocking down lncRNA-MM2P expression markedly inhibited M2 polarization and the macrophage-mediated promotion of tumorigenesis, tumor growth in vivo, and tumor angiogenesis [37]. Furthermore, a series of studies provided new insights into the regulatory mechanisms of dynamic transition between different macrophage phenotypes. miR-21 promotes macrophage repolarization from the M1 phenotype to the M2 phenotype [40, 41], while miRNA-23a/27a/24–2 promotes macrophage polarization from the M2 phenotype to the M1 phenotype [42]. Several recent studies elucidated that ncRNAs could be involved in macrophage polarization by regulating proinflammatory gene expression or influencing associated proteins interaction, such as lncRNAs Cox2, AK170409 and circHECTD1 [43–46].

Notably, the functions of macrophages in tumors need to be contextualized within the specific microenvironment. When macrophages express different levels of cytokines, they may function as anti-tumorigenic or pro-tumorigenic [47]. Nevertheless, our appreciation of the ability of ncRNAs to control macrophage function in cancer just comes from individual ncRNAs. It is likely to be modulated by a combination of several ncRNAs, many molecules or signaling pathways. Hence it deserves further investigation to explore ncRNAs in regulating macrophage function in cancer within systemical networks of multi-factor and multi-step.

#### Natural killer cells

Natural killer (NK) cells are a critical component of innate immunity as they often provide defense against infections and mediate antitumor immune responses [48–51]. Increasing evidence indicates that ncRNAs have important roles in NK cell biology in the contexts of development, inflammation, and tumor surveillance (Fig. 1b). A study by He et al. suggested that several miRNAs in the circulation, including miR-122, miR-15b, miR-21 and miR-155, could activate NK cells through Toll-like receptor signaling, which protected mice from tumor development [51]. Among the miRNAs involved in NK/T-cell lymphomagenesis, miR-21 and miR-155 are aberrantly overexpressed and activate AKT signaling via downregulation of the expression of tumor suppressors, including phosphatase and tensin homologue (PTEN), programmed cell death 4 (PDCD4), and Src homology-2 domain-containing inositol 5-phosphatase 1 (SHIP1), in natural killer-cell lymphoma/leukemia [52]. In addition, studies have shown that lncRNAs and circRNAs can also be involved in regulating NK cells cytotoxicity. A recent study from Fang et al. reported that lncRNA GAS5 expression was downregulated in NK cells from patients with liver cancer and lncRNA GAS5 inhibition suppressed NK cell cytotoxicity and promoted tumor growth. Overexpression of lncRNA GAS5 decreased miR-544 expression and increased RUNX3 expression, IFN-γ secretion and NK cell cytotoxicity [53]. It has been reported that lncRNA SNHG12 is a direct transcriptional target of c-Myc and that c-Myc-mediated upregulation of lncRNA SNHG12 expression promotes proliferation and inhibits sensitivity to cisplatin (CDDP) in natural killer/T-cell lymphoma [54]. In parallel, Ma and colleagues demonstrated that circRNA of AR-suppressed PABPC1 91 bp enhanced the cytotoxicity of natural killer cells against hepatocellular carcinoma. Subsequent mechanistic research revealed that upregulating UL16 binding protein 1 (ULBP1) expression in hepatocellular carcinoma cells led to enhanced innate immune surveillance by strengthening the cytotoxicity of NK cells [55].

ncRNAs are also involved in negative regulation of NK cell cytotoxicity. Membrane-bound MICA/B proteins are ligands of the natural killer group 2 member D (NKG2D) receptor found on natural killer (NK) cells, γδ(+) T cells and CD8(+) T cells [56, 57]. They could stimulate NKG2D to mediate NK cells recognition and elimination of virus-infected or neoplastically transformed cells [58, 59]. Members of the miR-17-92 cluster downregulated the expression of MICA/B and ULBP2 by targeting the MICA/B 3′-untranslated region (3′-UTR) and inhibiting the MAPK/ERK signaling pathway, respectively. Silencing these miRNAs enhanced natural killer cell-mediated cytotoxicity in breast cancer [60]. Besides, Xie et al. found that miR-20a-mediated MICA/B expression reduction inhibited NK cell cytotoxicity and promoted ovarian tumor proliferation and metastasis [61]. Yang et al. illustrated that histone deacetylase inhibitors epigenetically upregulated MICA expression by regulating the expression of the miR-17-92 cluster and MCM7 in hepatoma and enhanced the sensitivity of HCC to natural killer cell-mediated lysis [62]. These studies verified potential role of miR-17-92 cluster for promoting immune escape. Similarly, it has been reported that miR-10b shared the same mechanism in support of murine breast cancer escape from NK cell-mediated killing [63]. Besides, miR-146 [64], miR-150 [52, 65], and miR-30b [66] may function as tumor suppressors by suppressing pro-proliferative and pro-survival factors in NK/T cell lymphoma.

Since NK cell-mediated cytotoxicity plays an important role in enhancing immune function and suppressing immune escape, NK cell-associated therapy could be another emerging area of investigation. Considering the important effects of ncRNAs on NK cell functions, it is believed that ncRNAs may be efficacious therapeutic candidates. One of the possible reasons is that ncRNAs showed significant differential expression in cancer, which might provide insight into the roles of ncRNAs in regulating cancer progression. Moreover, NK cell must undgo rapid changes in response to immediately changed environment, thus ncRNAs are good choices to rapidly affect expression of associated genes within minutes without requiring new protein synthesis. In addition, somes ncRNAs have shown promise in pre-clinical and clinical development. Such as miRNAs mimics and small molecule agents (antisense oligonucleotides, locked nucleic acids, small interfering RNAs, etc.).

#### Neutrophils

It has been known for some time that neutrophils are involved in the tumor microenvironment. However, neutrophils are a heterogeneous population with both pro- and anti-tumor roles [67] . Given the important regulatory functions of miRNAs, the link between miRNAs and neutrophils has been explored. Landgraf et al. adopted miRNA cloning/sequencing approaches to explore miRNA profiles expressed of neutrophils [68]. Using a miRNA array Ma et al. found that the combination of miR-26a-2-3p and miR-574-3p had high specificity and sensitivity to differentiate neutrophil specimens of cancer patients from those of cancer-free controls [69] (Fig. 1c). These studies revealed that miRNAs could modulate neutrophil functions and cancer pathogenesis by regulating neutrophil gene expression. In parallel, Bazzoni et al. discovered a new set of miRNAs (miR-9, miR-187, miR-125a, miR-99b, and let-7e) induced by LPS in human polymorphonuclear neutrophils (PMNs) and monocytes [32]. Notably, among these miRNAs, only miR-9 is induced in neutrophils. It has been reported that ectopic expression of p50 contributes to LPS tolerance, chronic inflammatory conditions, and cancer under pathological conditions [70, 71]. Considering that NFkB1/p105/p50 is a target of miR-9, induction of miR-9 can suppress negative regulation by p50 homodimers in monocytes in systemic anti-inflammatory response syndrome and in cancer [32]. Wu and colleagues noted that high expression of the long noncoding RNA nuclear-enriched abundant transcript 1 (NEAT1) in whole blood was a novel diagnostic and prognostic biomarker of overall survival in colorectal cancer [72] (Fig. 1c). By separating the different immune cell types they identified that neutrophils had the highest expression of both NEAT1 variants and that NEAT1 expression in peripheral neutrophils might be the key parameter to distinguish CRC patients from healthy people [72].

### Roles of ncRNAs in adaptive immunity

**Fig. 2:**
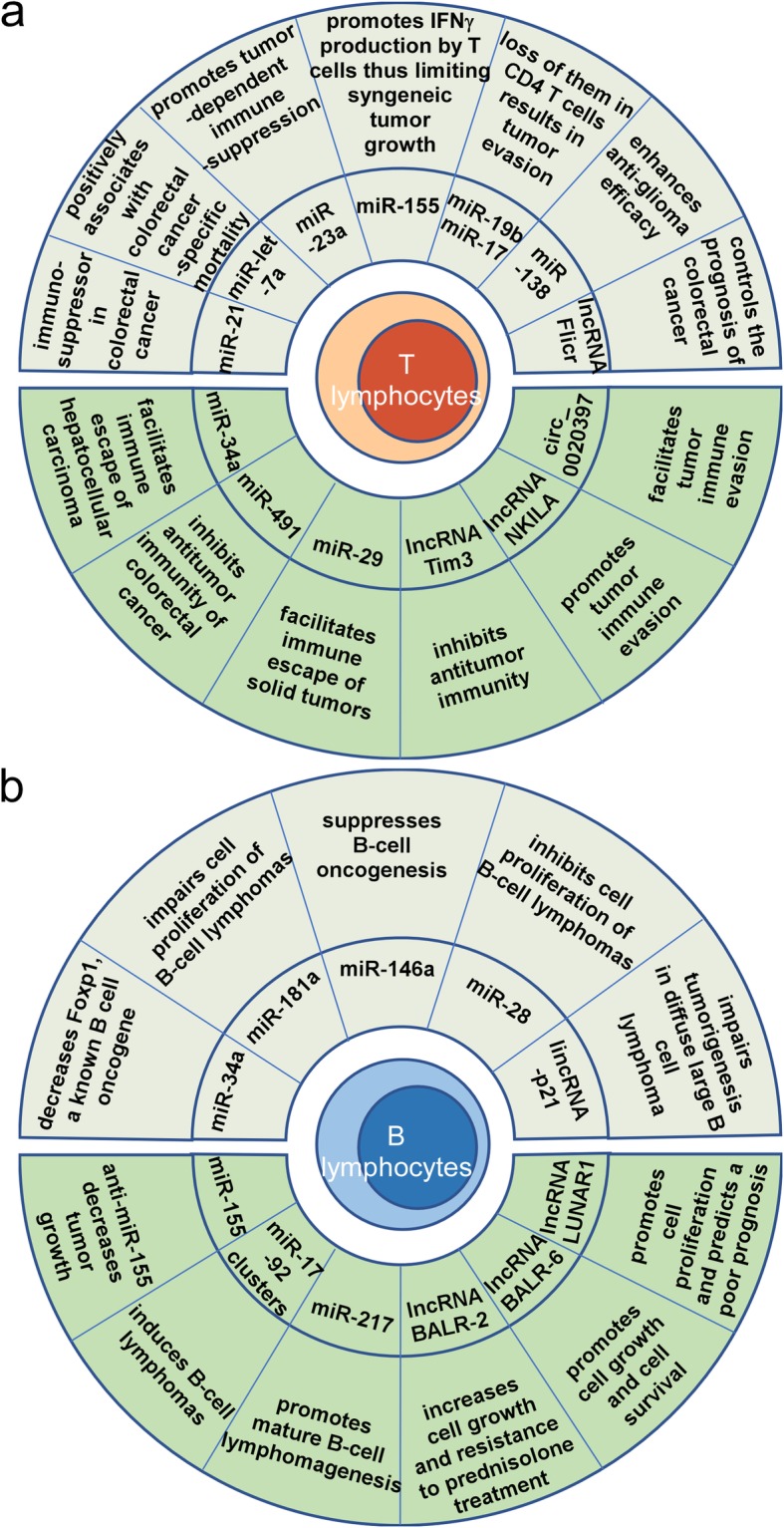
ncRNAs involved in adaptive immune regulation in cancer. **a** ncRNAs expressed in T cells modulate tumor immunity via diverse mechanisms. **b** ncRNAs function as important regulators of cancer immunity in B cells. The ncRNAs shown with a gray background are known to act as immunosuppressors, while the ncRNAs shown with a green background are known to act as immunopromoters

The adaptive immune response provides an extremely versatile mechanism of host organism defense and enhanced protection against subsequent exposure to the same antigen and/or reinfection with the same pathogen [73]. The adaptive arm of the immune system consists of B cells and T cells. Accumulating evidence indicates that ncRNAs in adaptive immunity influence tumor progression (Fig. 2).

#### T cells

T cells are a type of lymphocyte that is important to the adaptive immune system. They determine the specificity of the immune response to antigens (foreign substances) in the body. Emerging evidence suggest roles of ncRNAs in response to the antitumor T-cell-mediated adaptive immune response through a variety of regulatory mechanisms including controlling regulatory T cells (Tregs) functions, influencing T cell regulators, affecting immune checkpoints, regulating IFNγ production and so on (Fig. 2a). It is reported that miR-21 expression generally higher in colorectal cancer tissue than in paired normal tissue and was inversely associated with the densities of CD3^+^ and CD45RO^+^ T cells in colorectal cancer tissue [74]. Similarly, miR-21 was also shown to control the in situ expansion of CCR6(+) Tregs through the PTEN/AKT pathway in breast cancer [75]. Recently, it has been demonstrated that TGF-β-miR-34a-CCL22 signaling promotes Tregs recruitment, thus facilitating immune escape and enhancing venous metastasis of HBV-positive hepatocellular carcinoma [76]. LncRNAs Flicr fine-tunes the expression of Foxp3 influencing Tregs differentiation, stability, and function, thus associated the prognosis of colorectal cancers [77, 78]. BLIMP-1 is a promoter of cytotoxic T lymphocytes (CTLs) cytotoxicity and B7-H3 is a surface-expressed immunomodulatory glycoprotein that suppresses NK cells and T cells, both of which are important T cell regulators. miR-23a suppressed BLIMP-1 and correlated with impaired antitumor potential in patient, thus promoting tumor-dependent immunosuppression [79]. A study showed that miR-29 could downregulate B7-H3 to promote immune escape by solid tumors [80]. miR-138 exerts anti-glioma efficacy by targeting the immune checkpoints T-lymphocyte-associated antigen 4 (CTLA-4) and programmed cell death protein 1 (PD-1) [81]. A further study demonstrated that hsa_circ_0020397 acted as a sponge of miR-138 to promote the expression of telomerase reverse transcriptase and programmed death-ligand 1 (PD-L1), regulating the viability, apoptosis and invasion of colorectal cancer cells [82]. IFNγ is a multifunctional cytokine that plays a pivotal role in tumor immunity [83]. Huffaker et al. revealed that miR-155 promoted IFNγ expression by repressing SHIP1 leading to promotion of T cell-mediated antitumor immunity [84]. A subsequent study not only found that miR-155 expression within T cells was required to limit syngeneic tumor growth and promote IFNγ production by T cells but also revealed that immune checkpoint blockade could rescue microRNA-155-deficient induced immune escape [85]. miR-17-92 cluster has been reported to control Th1 responses through supporting IFN-γ production. Specifically, the loss of miR-17 and miR-19b, two members of the miR-17-92 cluster, in CD4^+^ T cells results in tumor evasion [86]. A study by Yu et al. reported that miR-491 could inhibit T cell proliferationan and IFN-γ production by targeting CDK4, TCF-1, and Bcl-xL in CD8^+^ T cells resulting in the inhibition of antitumor immunity [87]. Lnc-Tim3 that exhibits upregulated expression and is negatively correlated with IFN-γ and IL-2 production in tumor-infiltrating CD8^+^ T cells from hepatocellular carcinoma patients. Lnc-Tim3 promotes T cell exhaustion, which correlates with anti-tumor immunity [88]. Meanwhile, ncRNAs could also regulate T-cell-mediated adaptive immune response through other underlying mechanisms. For instance, miRNA let-7a expression might be inversely associated with T-cell densities in colorectal cancer tissue and positively associated with colorectal cancer-specific mortality, as reported by Dou et al. [89]. Another example is the lncRNA NKILA, which regulates T cell sensitivity to activation-induced cell death by inhibiting NF-κB activity. Calcium influx activates calmodulin removing deacetylase from the NKILA promoter and enhancing STAT1-mediated transcription, thereby influencing tumor immune evasion [90].

#### B cells

B lymphocytes (B cells), a type of lymphocyte white blood cell, are responsible for producing and secreting a particular immunoglobulin. miRNAs, key factors in various biological and pathological processes, have been reported to play immunomodulatory roles in B cells [91] (Fig. 2b). It has been suggested that miR-155 can act as an oncogenic miRNA in B-cell lymphoproliferative disorders and that its high expression correlates with a poor prognosis in lymphomas [92, 93]. A study by Medina et al. found that overexpressing miR-21 by Cre and Tet-off technologies led to pre-B lymphoma. These findings revealed the oncogenic role of miR-21 [94]. Some studies were performed to identify the correlation between miR-17-92 clusters and miR-217 in the promotion of B-cell lymphomas [95, 96]. In contrast, the findings of Rao et al. identified a role for miR-34a in connecting the p53 network with the suppression of Foxp1, a known B cell oncogene. Mechanistically, miR-34a could target the 3′-UTR of Foxp1 [97]. In parallel, miR-181a, miR-146a, and miR-28 are revealed as tumor suppressor genes [98–100].

In addition, many investigators studied the expression and function of lncRNAs in B cells, and evidence suggests that these ncRNAs might be protagonist in the pathogenesis of B-cell malignancies (Fig. 2b). It was reported that the lncRNAs BALR-2 and BALR-6 both promoted progression in B-acute lymphoblastic leukemia [101, 102]. Similarly, data have shown that in diffuse large B-cell lymphoma (DLBCL) LincRNA-p21 predicts a favorable clinical outcome and impairs tumorigenesis in diffuse large B-cell lymphoma patients treated with R-CHOP chemotherapy, whereas the lncRNA LUNAR1 is associated with cell proliferation and predicts a poor prognosis in diffuse large B-cell lymphoma [103, 104].

## Perspective: ncRNAs as potential targets for immunotherapies

**Fig. 3:**
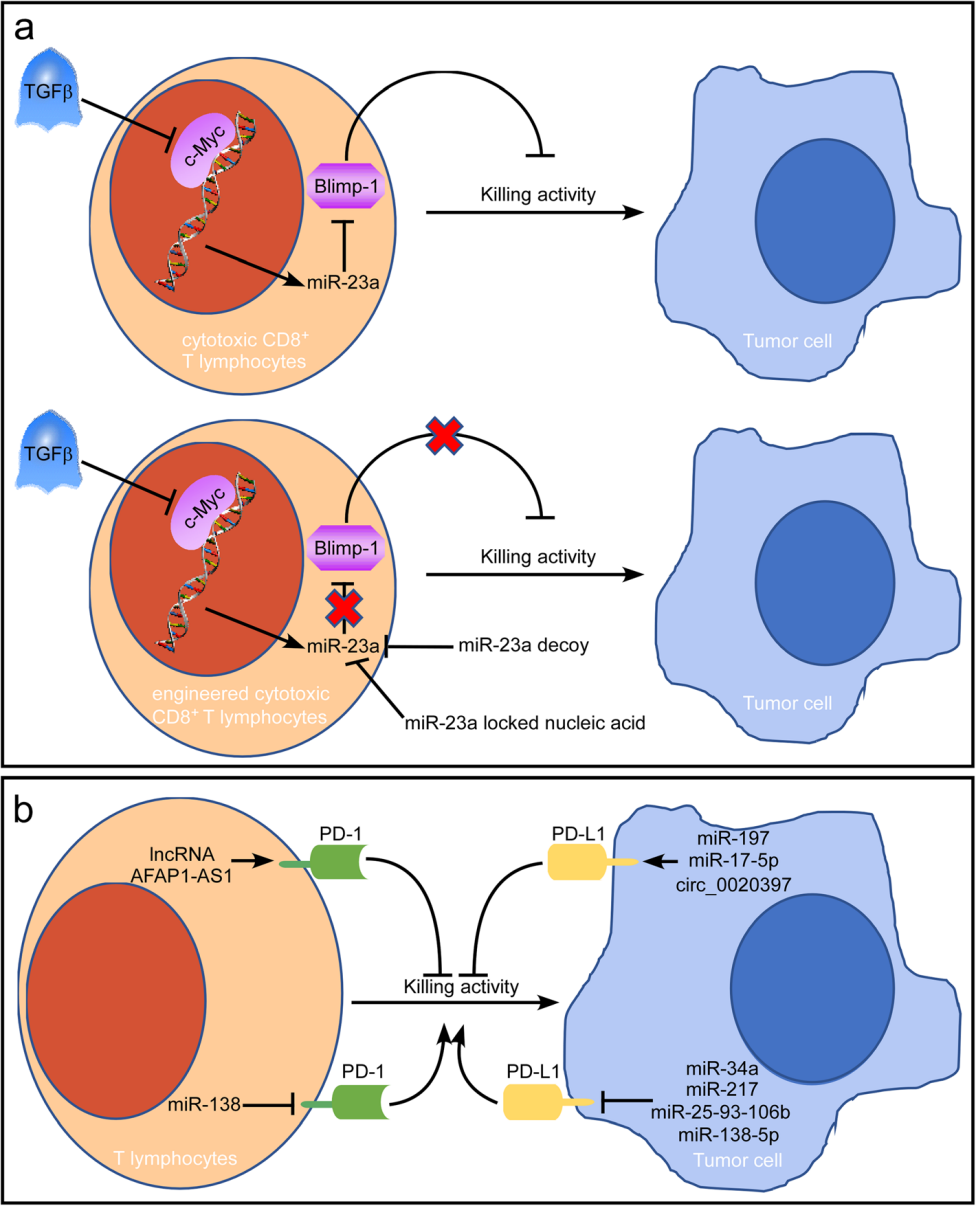
ncRNAs as potential targets for immunotherapies. **a** TGFβ represents one of the most significant immune barriers imposed by tumors. In the tumor microenvironment, TGFβ establishes immunosuppression via the c-Myc/miR-23a/Blimp-1 axis. In engineered cytotoxic CD8^+^ T lymphocytes blockade of miR-23a restores adoptive T cell transfer therapy. **b** Immune-checkpoint proteins, such as PD-1 and its ligand PD-L1, can tightly regulate the activation or repression of the functions of T cells and influence the cancer immune response. The most relevant ncRNAs invovled in regulating the PD-1/PD-L1 pathway are included in Fig. 3b

In the late 1990s, immunotherapy was introduced, and so far it has developed remarkable and durable responses in a subset of cancer patients. Currently, how to make more patients respond to immunotherapy becomes the major concern. Since the key roles of specific ncRNAs in the regulation of tumor immunity have been firmly established, researchers began to assess if targeting such ncRNAs could bring benefits to immunotherapy (Fig. 3).

CAR T-cell therapy is a new type of adoptive cell transfer immunotherapy. After harvesting T cells from a patient, researchers expanded the cells and/or genetically modified them in the laboratory. After these processes, the genetically modified T cells were returned to the patient to attack and potentially eliminate cancer cells [105]. Recent work by the Lin group showed that TGFβ inhibited c-Myc activity to release the transcriptional brakes on pri-miR-23a in cytotoxic CD8+ T lymphocytes and upregulated miR-23a suppressed expression of Blimp-1 leading to immunosuppression [106] (Fig. 3a). In engineered cytotoxic CD8+ T lymphocytes blockade of miR-23a restored adoptive T cell transfer therapy by miR-23a decoy or locked nucleic acid.

Another area of cancer immunotherapy in which we are making extraordinary progress is immune checkpoint blockade. Immune-checkpoint proteins are localized on the surface of T cells and function as brakes to stop attacks on tumor cells when triggered by high levels of proteins expressed by some tumors [107]. Immune-checkpoint proteins, such as cytotoxic CTLA-4, T-cell membrane protein 3 (TIM3), lymphocyte activation gene 3 (LAG3), B- and T-lymphocyte attenuator (BTLA), and PD-1 and the PD-1 ligand PD-L1, can tightly regulate the activation or repression of functions of T cells [108]. In particular, monoclonal antibodies targeting the PD-1/PD-L1 pathway have made substantial breakthroughs in immune checkpoint therapy [109]. High levels of PD-1 and its ligand PD-L1 are associated with a poor prognosis in several cancers, as overactivation of the PD-1/PD-L1 pathway results in suppression of the anticancer immune response [108] (Fig. 3b). As described earlier, miR-138 exerts anti-glioma efficacy by targeting the immune checkpoint molecules CTLA-4 and PD-1 by inhibiting tumor-infiltrating Tregs [81]. In addition, miR-34a [110, 111], miR-138-5p [112], miR-25-93-106b [113], and miR-217 [114] have been demonstrated to inhibit the expression of PD-L1, thereby increasing antitumor immunity and inhibiting multiple metastatic traits. In contrast, modulation of miR-17-5p [115] and miR-197 [116] has been shown to be crucial for protumor function. Other types of ncRNAs, including lncRNAs and circRNAs, have also been reported to be involved in the regulation of the PD-1/PD-L1 pathway. Tang et al. identified that the lncRNA AFAP1-AS1 was significantly correlated with PD-1 in nasopharyngeal carcinoma and that their coexpression predicted a poor prognosis [117]. This study revealed that the lncRNA AFAP1-AS1 was a potential target for future clinical trials of anti-PD-1 immunotherapies. In another recent study, a group showed that hsa_circ_0020397, a sponge of miR-138, promoted the expression of TERT and PD-L1, regulating the viability, apoptosis and invasion of colorectal cancer cells [82].

Although advances made in the immunotherapies of cancer, many tumors remain incurable because of multiple mechanisms of immune resistance. It is of critical importance to understand additional biological properties that are significant modulators of therapeutic response. As discussed before, ncRNAs contribute to the regulation of tumor immunity by influencing many biological processes, including macrophage polarization, NK cell cytotoxicity, T cell responses, etc. Therefore, ncRNAs may be efficacious therapeutic targets for tumor immunotherapies. For example, recent studies reveal that blockade of miR-23a restored adoptive T cell transfer therapy providing new insights into improvement of immunotherapy efficacy [106]. Meanwhile, ncRNAs involved in the regulation of the PD-1/PD-L1 pathway may have a major role in limiting effective cancer immunity [110–117].

One major concern is how we could efficiently and safely target these ncRNAs which show great regulatory functions in immune cells or cancer cells. It is worth noting that recently many new technologies and approaches in RNA biology are created. For example, in addition to miRNA mimics or inhibitors, Target Site Blocker (TSB) is a novel way for targeting miRNAs. LNA-TSB is LNA-enhanced antisense oligonucleotides that bind to the mRNA target site of a miRNA, thereby preventing miRNAs from gaining access to that site, which allow researchers to investigate the effects of the miRNA on a single endogenous target [118]. Importantly, all these oligonucleotides, including miRNA mimics, inhibitors, and TSB could be used for in vivo delivery after specific modification, which allows researchers to assess the therapeutic effects on mice, monkeys, or other mammals. Although some studies have validated the therapeutic effects of targeting miRNAs in vivo [119], it is still a huge gap between animal analysis and clinic application. According to our knowledge, there is only one clinical trial about miRNA therapy, MRX34 Liposomal Injection, and unfortunately it was just terminated because of toxicity [120]. Since miRNAs are always generally expressed in most types of cells, systemic delivery of miRNA associated drugs may induce many side effects or toxicities. Thus, how to precisely deliver miRNA associated drugs to cancer cells or immune cells becomes a critical task. Other questions should also be considered, such as: what kinds of patients are suitable for miRNA based therapy? How to combine miRNA drugs with immunotherapy?

Another concern is thus far only a small portion of lncRNAs and circRNAs has been studied. Unlike miRNA that is broadly expressed in most of cells, some lncRNAs, such as PCA3, are specifically expressed in cancer cells [121]. This may make them good targets for novel therapy. LNA enhanced antisense oligonucleotides (LNA-ASO) has shown optimal effects on targeting lncRNAs, especially for those are localized in nucleus, and it could also be used for in vivo delivery [122]. Thus, it is of great importance to identify more immune cell or cancer cell specific lncRNAs and clarify their regulatory mechanism, so that researchers can further assess their therapeutic potential in vivo and thus develop new drugs.

In summary, it is no doubt that the better understanding of the ncRNAs regulatory roles in cancer immunity in the recent years will largely improve the efficiency of immunotherapy in the future. Continued efforts to uncover the roles of all kinds of ncRNAs in tumor immunity will pave the way for even improved understanding, prevention, and cancer treatment and enable immunotherapy to be adjusted and made more consistent with a patient’s biological characteristics.

## Data Availability

Not applicable.

## Abbreviations

3′-UTR: 3′-Untranslated region
BTLA: B- and T-lymphocyte attenuator
circRNAs: Circular RNAs
CTL: Cytotoxic T lymphocyte
CTLA-4: Cytotoxic T-lymphocyte-associated antigen 4
DCs: Dendritic cells
DLBCL: Diffuse large B-cell lymphoma
LAG3: Lymphocyte activation gene 3
lncRNAs: Long non-coding RNAs
miRNAs: Micrornas
ncRNAs: Non-coding RNAs
NEAT1: Nuclear-enriched abundant transcript 1
NK cells: Natural killer cells
NKG2D: Natural killer group 2 member D
PD-1: Programmed cell death protein 1
PDCD4: Programmed cell death 4
PD-L1: Programmed death-ligand 1
PMN: Polymorphonuclear neutrophils
PTEN: Phosphatase and tensin homologue
SHIP1: Src homology-2 domain-containing inositol 5-phosphatase 1
TAMs: Tumor-associated macrophages
TIM3: T-cell membrane protein 3
Tregs: Regulatory T cells
ULBP1: UL16 binding protein 1
